# Time-Dependent Structure Assessment of Conjugated Polymer Aggregates in Solution by Single-Molecule Fluorescence Spectroscopy

**DOI:** 10.1021/acs.macromol.5c02031

**Published:** 2026-01-21

**Authors:** Esther Schäfer, Michael Sommer, Maria Ott

**Affiliations:** † Institute for Chemistry, 38869 Chemnitz University of Technology, Str. der Nationen 62, 09111 Chemnitz, Germany; ‡ Institute of Biochemistry and Biotechnology, Martin Luther University Halle-Wittenberg, Protein Biochemistry, Kurt-Mothes-Str. 3, 06120 Halle (Saale), Germany; § Forschungszentrum MAIN, Chemnitz University of Technology, Rosenbergstraße 6, 09126 Chemnitz, Germany

## Abstract

Monitoring and understanding the aggregation kinetics of n-type polymers provide strategies to favorably control aggregation and thereby optimize conjugated polymers ink shelf life, printability, and thin-film properties. Here, the in situ characterization of n-type copolymer aggregates employing ensemble absorbance and fluorescence spectroscopy for spectral characterization, in combination with single-molecule fluorescence spectroscopy methods to identify subcategories of aggregates, is reported. Specifically, we utilize a diffusion-based single-molecule burst method that resolves individual aggregates as they traverse the observation volume, allowing us to determine the aggregate size, concentration, and chain conformation through the statistical analysis of single-aggregate fluorescence data. Base-stable P­(EO-NDIT2) with branched ether-based side chains self-assembles from molecularly dissolved chains into nano- to micrometer-sized aggregates over the course of weeks to months, depending on the solvent used. Through spectral decomposition and polarization-sensitive single-molecule fluorescence spectroscopy, the aggregates were categorized by their size into small (*R*_h_ approximately 60 nm) and large (*R*_h_ approximately 300 nm) aggregates and monitored with time. An increase in size was correlated with enhanced fluorescence brightness and red-shifted emission as well as an increase in internal order, as revealed by emission anisotropy. This increase in order within the aggregates may be related to alignment of crystalline domains and a planarization of the polymer backbone torsion.

## Graphical abstract

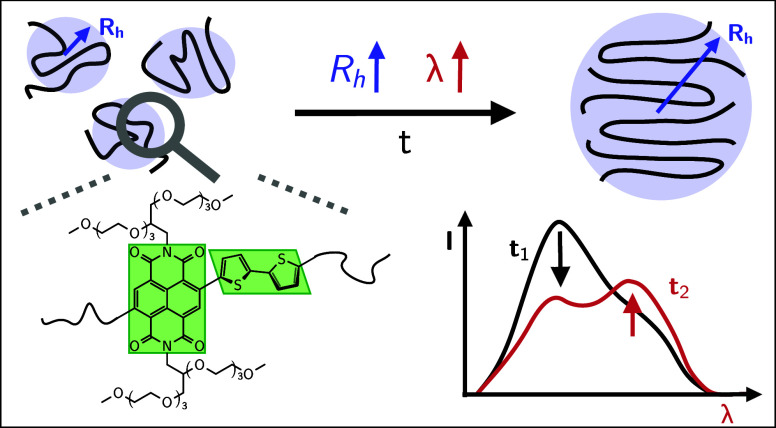

## Introduction

The aggregation behavior of conjugated polymers in solution directly propagates into the performance of printable organic electronic devices, such as organic solar cells and organic electrochemical transistors.^1–5^ Precise monitoring and understanding of the aggregation kinetics of conjugated polymers to control film morphology through solution processing and deposition in the ink preparation and printing process is crucial for achieving high electrical performance.^1,6–8^ Enhancing this electrical performance is essential for the integration of these organic electronic devices into everyday use. The extent of aggregation depends first and foremost on the structure and the side chains of conjugated polymers.^9–11^ For a given conjugated polymer, solvent, concentration, temperature, and time, all play a role in aggregation in solution, and thus, preparation pathways determine thin-film microstructures.^1,12^ Different microstructures, in turn, alter the optical, thermal, mechanical, and electrical properties of semiconductor thin films.^13,14^ In the context of printing, aggregates acting as colloidal particles affect ink properties, such as viscosity, vapor pressure, and interface interactions, including the contact angle. These effects can lead to issues like nozzle clotting.^15,16^ Time-dependent aggregation processes degrade the ink quality and shorten the ink shelf life, which is a key challenge for commercialization.^17–19^ Monitoring and understanding the aggregation kinetics of conjugated n-type polymers offer strategies to control aggregation favorably, thus optimizing ink shelf life, printability, and thin-film properties.

N-Type semiconducting polymers, composed of naphthalene-1,4,5,8-bis­(dicarboximide) (NDI) and 2,2′-bithiophene (T2) building blocks, as poly­{[*N,N*′-bis­(2-octyl-dodecyl)-naphthalene-1,4,5,8-bis­(dicarboximide)-2,6-diyl]-*alt*-5,5′-(2,2′-bithiophene)} (P­(OD-NDIT2)), first introduced by Guo and Watson^20^ and Facchetti et al.,^21^ have since become benchmark materials for various organic electronic applications.^5,22,23^ Side chain engineering, particularly using oligoethylenoxide (EO), has resulted in devices that are stable in the presence of water^24,25^ and has enhanced thermoelectric performance through improved dopant miscibility and loading.^22,26,27^

Poly­{[*N,N*′-2,3-bis­(methoxy­(triethylenoxide))­propane-naphthalene-1,4,5,8-bis­(dicarboximide)-2,6-diyl]-*alt*-5,5′-(2,2′-bithiophene)} (P­(EO-NDIT2)) (see Figure 1a) is one such side-chain-engineered NDI-T2 copolymer with purely ether-based, base-stable side chains. The polymer exhibits time-delayed aggregation over several days to months, depending on the solvent used.^28^ Aggregation is a characteristic behavior observed in conjugated polymer solutions.^29–33^ This process is widely monitored by optical spectroscopy, which typically does not yield insight if these time-dependent processes are due to the formation of new aggregates or changes in existing ones, such as restructuring and growth. Single-molecule methods of conjugated polymers offer a unique opportunity to study chain conformation and aggregation, as demonstrated by polarization-sensitive fluorescence imaging of chains embedded in nonfluorescent films.^34–37^ In particular, these experiments could demonstrate the correlation of spectral features with backbone configurations,^38^ intramolecular interactions of single chains,^37,39^ and interchain morphologies of aggregates.^36^

**Figure 1:**
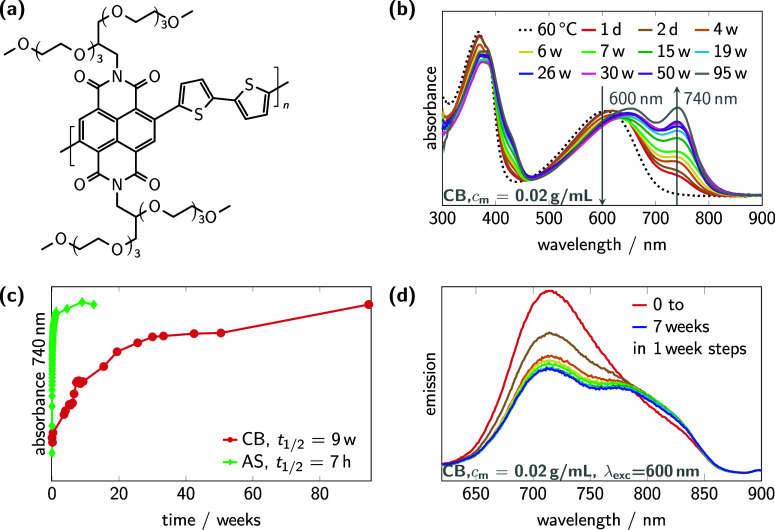
Time-dependent absorbance and emission spectroscopy of P­(EO-NDIT2) solutions (mass concentration (*c*_m_) = 0.02 g mL^–1^). (a) Structure of P­(EO-NDIT2). (b) UV/vis spectra of a polymer solution during thermal annealing (dotted line) and up to 95 weeks (solid lines) at room temperature. (c) The plateau of equilibration in AS (green) is reached within several days, whereas in CB (red), several months are required. (d) The emission spectra of polymer solutions in CB were monitored over a period of 7 weeks at an excitation wavelength of 600 nm. The results showed a decrease in the concentration of molecularly dissolved chains with an emission peak at 720 nm, favoring the formation of aggregated species that emit at a red-shifted wavelength of 750–850 nm. For spectra at the excitation wavelength of 740 nm, see Figure S3.

In solutions, single-molecule fluorescence spectroscopy (smFS) convinces by providing aggregate size distributions, as opposed to commonly used in situ methods like small-angle X-ray scattering, small-angle neutron scattering, and sedimentation velocity experiments^29^ which typically yield only an averaged radius. Dynamic light scattering, another common technique for particle size determination that also provides size distribution data, is unsuitable for studying this polymer. This limitation arises because substances with strong absorption in the laser’s spectral range and strong fluorescence emission compromise data quality.^40^ In addition to offering insight into the size distribution of aggregates, smFS allow the determination of aggregate concentrations and enable conclusions regarding local chain environments.^41,42^

In this work, we apply the method of burst analysis spectroscopy (BAS) from smFS to investigate the time-dependent aggregation kinetics of P­(EO-NDIT2). By spectrally discriminating aggregates from the nonaggregated chains, we identify them as red-shifted emitters, consistent with ultraviolet–visible (UV/vis) absorbance and fluorescence emission spectroscopy results. Application of our BAS method allows for the grouping of the aggregates by size into aggregate categories. Our analysis reveals that in anisole (AS), small aggregates form first and remain constant in size and concentration with time, while larger aggregates emerge as a secondary process. In chlorobenzene (CB), small aggregates initially form and increase in concentration but later reorganize into structures of larger size. Notably, our determined aggregate sizes are consistent with the results from optical microscopy and atomic force microscopy (AFM). As aggregation progresses, the accompanying changes in polarized emission could be interpreted as alignment of crystalline domains combined with increased backbone planarization due to denser chain packing and π–π interactions. This structural evolution is accompanied by a red-shift in the fluorescence emission, linking optical properties to aggregate morphology. Thus, this method offers a comprehensive and in situ characterization of fluorescent conjugated polymer aggregates, providing valuable insights into their aggregation behavior. The presented methodology and findings significantly advance our understanding of aggregation processes, enabling the development of targeted strategies to optimize printability and thin-film properties in n-type polymer applications.

## Results and Discussion

### Time- and Temperature-Dependent Spectroscopic Features of P­(EO-NDIT2)

The aggregation process of P­(EO-NDIT2) in solutions of AS and CB is monitored by UV/vis absorbance and fluorescence emission spectroscopy, as shown in Figure 1b–d. The spectroscopic properties of the material P­(EO-NDIT2) will be discussed in the following section, providing a basis for a comprehensive evaluation of the aggregation process using fluorescence correlation spectroscopy (FCS) and smFS.

The **absorbance spectra** in Figure 1b enable the differentiation between solutions with fully dissolved and partially aggregated polymer. The nonaggregated solution of P­(EO-NDIT2) in CB at 60 °C shows only a high-energy peak between 300 and 400 nm attributed to the π–π* transition, as well as a broad, low-energy peak from 500 to 700 nm, corresponding to the charge transfer (CT) transition. Upon cooling to room temperature and aging at ambient conditions, an additional CT band between 650 nm and 850nm appears. This red-shifted band indicates the formation of aggregated species through self-assembly of the polymer chains into ordered, most probably π-stacked aggregates. In AS, the polymer is also prone to aggregation, showing identical spectral features as discussed for CB. In contrast to AS and CB, the polymer remains dissolved in chloroform (CHCl_3_) without aggregation over months (see UV/vis spectra in Figure S2a).

In general, spectral shifts of aggregated chromophores arise from a sensitive interplay between long-range Coulomb coupling and short-range CT-mediated coupling, which are very sensitive to variations of the chain’s architecture and packing.^43–46^ The resulting spectral properties can be attributed to vibronic coupling and intermolecular CT, modified by nonresonant dispersion interactions as well as other effects such as aggregation-induced planarization of the backbone.^47^ In the case of P­(OD-NDIT2), the low-energy band structures at 800 nm were assigned to chain segregation and packing by H-like aggregate patterns with strong intermolecular coupling among excited states.^48^ Density functional theory (DFT) calculations of methylated NDI-T2 trimers further revealed that the red-shifted emission may not only be due to π–π stacking but also due to a planarization of the torsion angle between the donor (T2) and acceptor (NDI) units upon stacking.^29^ However, these signatures might be further influenced by the chain architecture, including regio-regularity^49^ or side chain modifications,^50^ which complicates a straightforward interpretation of the results presented in this study.

The time dependence of the aggregation process can be quantified by tracking the absorbance at the aggregation peak maximum of 740 nm, as shown in Figure 1c, revealing the slow equilibration of aggregate formation by increasing the total mass of aggregates and depletion of nonaggregated chains at room temperature. In CB, equilibration takes several months with a half time (*t*_1/2_) of 9 weeks, while in AS, the aggregation stabilizes within several days, with *t*_1/2_ of 7 h. The markedly slower aggregation in CB compared to AS might be attributed to the difference in solvent polarity indicated by the dielectric constants of 5.6 and 4.3, respectively.^51^ CB thus dissolves the polymer more effectively and delays aggregation. This interpretation is also consistent with recent findings linking a change from unpolar aliphatic to polar ethylene glycol side chains to a change in the polar part of the Hansen solubility parameter of conjugated polymers.^52^

The **emission spectra** in Figure 1d of polymer solutions in CB were taken at room temperature in weekly intervals over seven weeks, with excitation of the CT transition at 600 nm after previous thermal annealing of the sample. The broad emission band at 650–800 nm peaked at 720 nm, attributable to molecularly dissolved chains, decreases with time. Concurrently, aggregated species emitting in the range of 750–850 nm were formed. This red-shifted emission is consistent with the red-shifted absorbance observed in UV/vis spectroscopy in Figure 1b. Upon selective excitation of the CT transition of the aggregates at 740 nm, as shown in Figure S3, fluorescence is only observed in solvents prone to aggregation, while no emission is detected in good solvents, such as CHCl_3_. Hence, this red-shifted aggregate absorbance and emission enables spectral distinction of the aggregates from the emission of molecularly dissolved, nonaggregated chains.

### Fluorescence Correlation Spectroscopy (FCS)

The FCS measurement is performed using a confocal microscope setup, as illustrated in Figure 2a, in which only the so-called confocal volume, an ellipsoid region of approximately 0.15 fL in volume, is probed. This small volume with a full width at half-maximum (fwhm)-diameter of about 430 nm just contains a small subset of the polymer solution, minimizing background interference.^53^ The diffusion of the chromophores into and out of the confocal volume results in temporal fluctuations in photon emission, as displayed in Figure 2b. These intensity time traces are employed to calculate correlation functions using well-established FCS methods (see Experimental Section). The particle size information, which is directly related to the characteristic decay time of the correlation function, is obtained by curve fitting, as shown in Figure 2c. The dwell time (τ_D_) in FCS thereby correlates to the hydrodynamic radius (*R*_h_) by eqs 1 and 2 with τ_D_ ∼ *R*_h_. For polymer solutions at room temperature in CB and AS, a two-component correlation analysis was applied, revealing 7(1.5) nm for (most likely) molecularly dissolved chains and of 65(27) nm for early aggregates. The large standard deviation of the latter is due to the small amplitude of the aggregate fraction, which is caused by lower concentrations compared to nonaggregates. These measurements were performed at room temperature after thermal annealing, ensuring molecular dissolution of the polymer prior to the experiment. Such samples are subsequently referred to as fresh solutions. The following time-dependent increase in the decay time for P­(EO-NDIT2) in CB, shown in Figure 2c, directly relates to a reduced diffusion coefficient (D) and hence an increase in size. The additional increase in size after 1 week displays the formation of even larger aggregates due to slow secondary processes.

**Figure 2:**
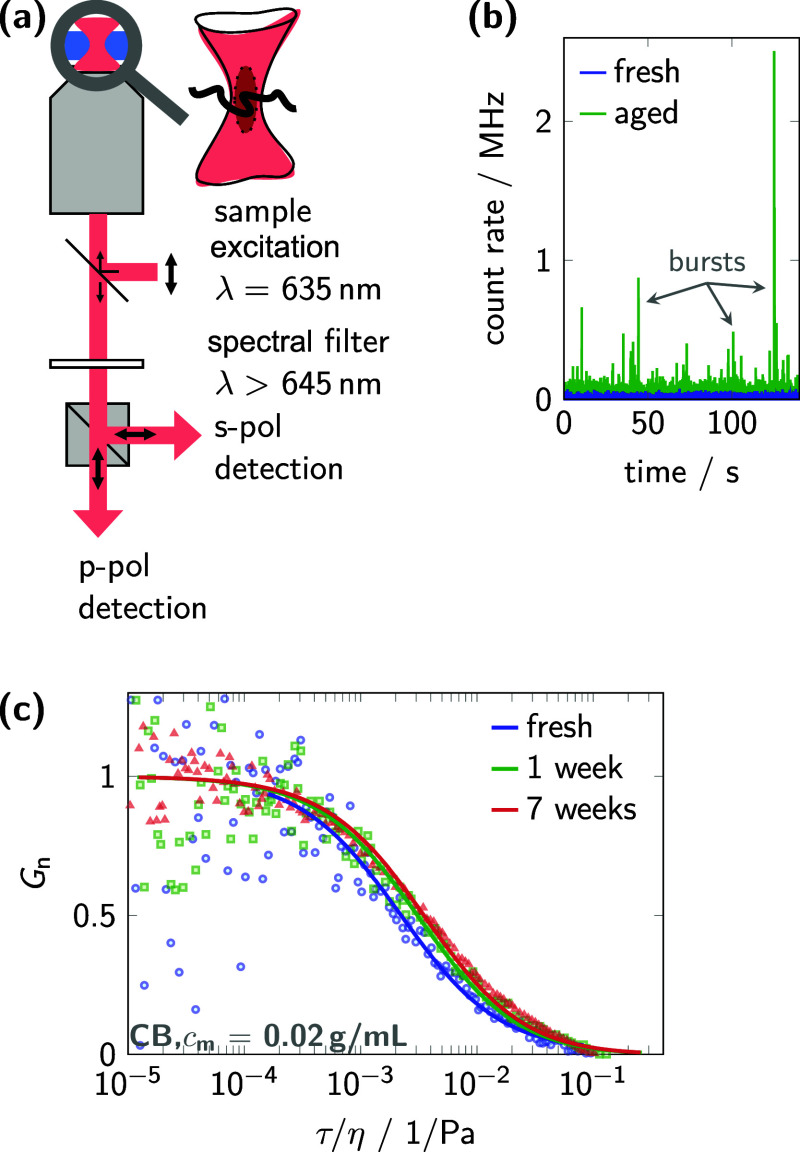
Microscope setup for polarization-sensitive experiments and data analysis of FCS experiments. (a) The scheme of the inverted microscope setup with the probed ellipsoid confocal volume is shown, which was used for FCS and polarization-sensitive smFS experiments. (b) The obtained fluorescence time traces for a fresh sample with low photon yield and no apparent bursts are shown in blue, while the time traces for an aged and aggregated sample with significant photon bursts are shown in green. (c) Time-correlation analyses of the detected emission photon flux result in normalized correlation functions (*G*_n_(τ/η), symbols), which allow the determination of ensemble particle sizes at different solution ages by curve fitting (lines, for details see Experimental Section). The increasing mismatch between the simple fitting curve and the correlation data of samples with increasing age indicates a growing amount and dispersity of aggregates.

### Microscopic Characterization of Aggregates

The generation of aggregates as seen in UV/vis spectroscopy and FCS is further supported by interference contrast (ICR) optical microscopy and AFM observations of a 14 week-aged AS sample, as shown in Figure 3. Optical microscopy in Figure 3a reveals many small and a few large, bright aggregates, which AFM analysis in Figure 3b characterizes as having heights between 100 and 300 nm and diameters about 5 μm. In addition to these large aggregates, most aggregates in Figure 3a appear as small dark-blue features in ICR microscopy. These small aggregates have a mean diameter of about 1.5 μm according to image autocorrelation analysis of regions in which only small aggregates were present.^54,55^ Small aggregates are also distinctly visible in the AFM phase image in Figure S5.

**Figure 3:**
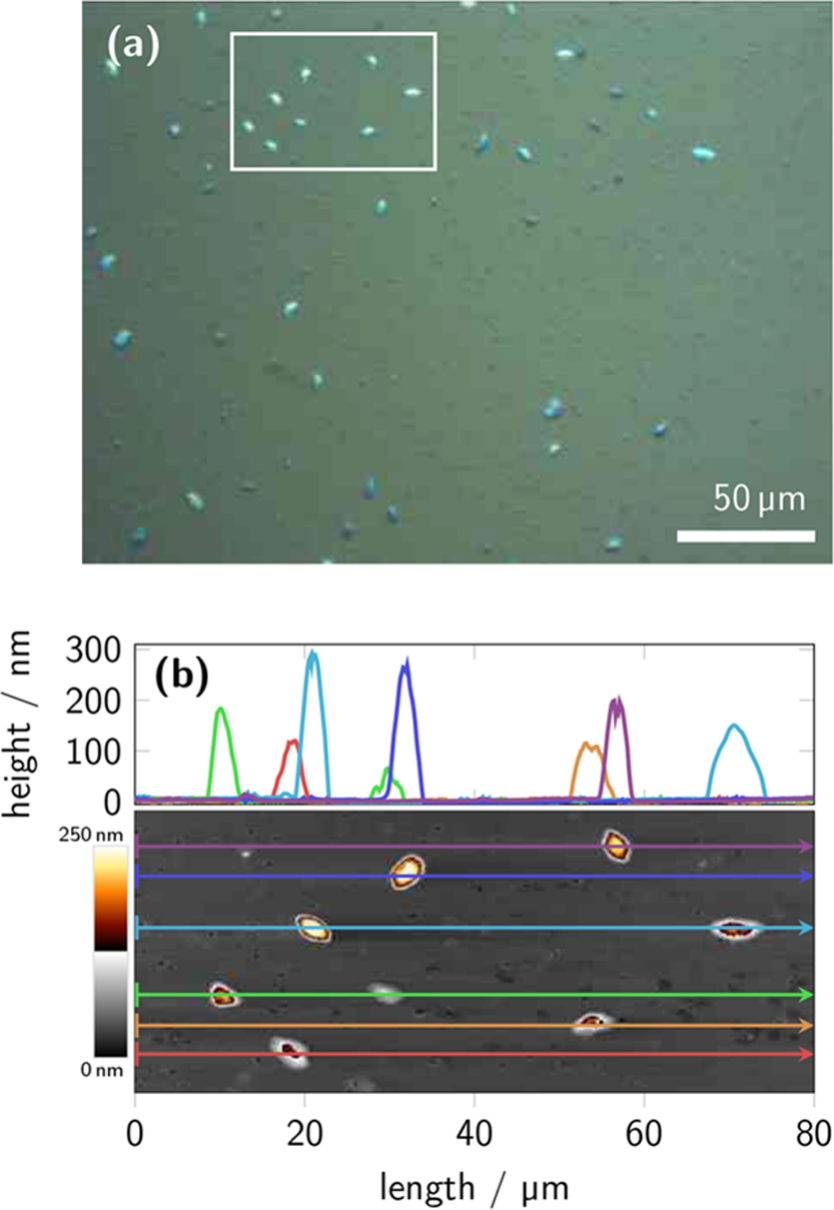
Microscopy of a drop-cast P­(EO-NDIT2) sample (0.08 g L^–1^, AS solution) after 14 weeks, showing (a) an optical microscopy image in ICR mode with large, bright, and small, dark-blue features and (b) an AFM height image of the region indicated in the optical microscopy image (white frame), including height profiles.

### Characteristics of Single Aggregates by smFS

Given the heterogeneous aggregate size, FCS analysis is no longer a suitable method. Therefore, we instead applied the tools of smFS, which are valid for very low concentrations of bright particles, a criterion that is well met for the observed aggregates.^42^ The concentration of the aggregates should be below the critical concentration of 6 × 10^18^ particles/L, which corresponds to the probability of having an average of 0.1 particles within the confocal volume.^53^ At these concentrations, burst analysis spectroscopy (BAS) methods in smFS enable multiparameter parametrization of single aggregates, allowing the identification and grouping of aggregates into categories of distinct entities within heterogeneous samples. The characterization is conducted with regard to the following parameters: the average passage time is related to size, molecular brightness is related to aggregate number or mass, and fluorescence anisotropy is related to molecular chain arrangements, as exemplified by the polymer backbone (see Experimental Section for more details). The molecular fluorescence anisotropy can be obtained using a polarization-sensitive setup, as illustrated in Figure 2a, allowing the detection of photons depending on their polarization.

### Spectral Analysis of Aggregates

Initially, we aimed to verify whether the observed fluorescence bursts, as depicted in Figure 2b, are indeed related to aggregates. As aggregates are expected to exhibit a red-shifted emission spectrum (as previously discussed about Figure 1), fluorescence bursts should be spectrally distinguishable from the less fluctuating background. However, if the less fluctuating regions exhibit the same spectral characteristics as the larger fluorescence bursts, the smFS approach would fail to reveal significant features of aggregates. Therefore, we analyzed the fluorescence intensities after adding a set of optical filters that provide spectral distinction of photons with 645 nm < λ < 775 nm and with λ > 775 nm, as shown in Figure 4a. These experiments were conducted with 45° linear polarized light to suppress polarization-biased detection.

**Figure 4:**
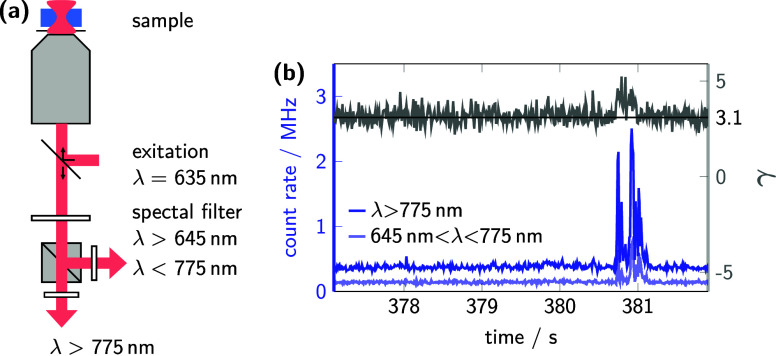
Microscope setup and data processing for spectral-distinct smFS. (a) The experimental setup, equipped with spectral filters that discriminate emitted photons by wavelength, enables the differentiation of aggregates and molecularly dissolved chains. (b) Time trace of a photon burst obtained in a measurement using the setup displayed in (a). The elevated γ value during the burst serves as an indicator of the spectral red-shift, confirming that the burst originates from an aggregate.

The emission maxima of P­(EO-NDIT2) solutions at different aging stages, shown in Figure 1d, suggest that photons with a wavelength below 775 nm correspond mostly to molecularly dissolved chains, while photons at higher wavelengths correspond mostly to aggregates. Despite some overlap of the broad fluorescence bands, the spectral filtering enables sufficient differentiation between the two species by determination of the spectral shift (see Figure S2b). In order to monitor the spectral shift, we further defined a red-shift intensity ratio (γ) by γ = *I*_λ>775nm_/*I*_λ<775nm_.

Applying this procedure of spectral decomposition to an aged sample containing aggregates, two time traces were generated, as shown in Figure 4b. Notably, the number of the detected photons differed significantly during strong fluctuations, as illustrated by the representative photon counts, particularly during the representative burst at 381 s. The parameter γ revealed a distinct red-shift for the burst region with an average γ of 4.0 compared to adjacent regions that contain small fluctuations and a γ of 3.1. This finding confirms the previously anticipated link between the large fluorescence bursts from aggregates and red-shifted fluorescence, as shown in Figure 1.

### Aggregates in AS Solutions

#### Burst Selection

In smFS, each single photon burst, representing a single aggregate, is individually characterized, enabling the display of the sample’s heterogeneity. To establish criteria for categorizing aggregate sizes, we selected the apparent molecular dwell time (τ_d_) and molecular brightness (MB) as suitable parameters. A category average of τ_d_, τ_D_ = ⟨τ_d_⟩, can be considered to be directly proportional to size, which in our case is the apparent *R*_h_. This relationship allows us to relate smFS to FCS findings of the previous section, as detailed in the Experimental Section. It is worth noting that our *R*_h_ values are based on the assumption of spherical particles. Consequently, in the case of elongated particles, the size of the aggregates is slightly overestimated. For example, an ellipsoidal particle with an axial ratio of 1:2 would result in a 10% reduced diffusion coefficient, leading to a 10% larger apparent *R*_h_, compared to a sphere.^56^ However, compared to the scale of the observed changes, these shape-related deviations play only a minor role.

Figure 5 shows burst maps of a fresh AS sample as well as after one and seven weeks of aging at room temperature. Immediately after thermal annealing, the lower left corner of the plot is densely populated with bursts associated with the category of so-called small aggregates, characterized by low τ_d_ and low MB values, defined by the number of photons (*N*_phot_) with MB = *N*_phot_/τ_d_. Bursts in the bottom right area are results of the broad distribution of photon counts^57^ and hence do not represent a different category of particles. After one and seven weeks, the bursts appear increasingly scattered toward the top right of the plot with long τ_d_ and high MB values. Our analysis allows us to cluster these bursts into distinct categories, one representing the larger aggregates (green symbols) and the other the dominant fraction of smaller aggregates (blue symbols). The observation of these two categories of aggregates is consistent with the previous findings revealed by microscopy in Figure 3. Our method, based on the discrimination criteria described in the Experimental Section, allows for the identification of distinct aggregate populations and the detailed monitoring of their formation. This differentiated examination is essential, as it highlights the limitations of relying solely on average values of the entire ensemble, which may mask the presence of smaller subgroups in dispersed samples, as demonstrated in this study.

**Figure 5:**
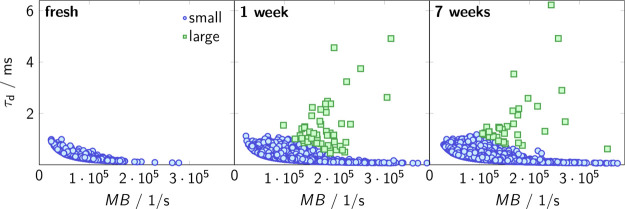
Time-dependent single-aggregate characteristics in AS solutions: Results of the discriminant analysis of aggregates regarding diffusion time, τ_d_, and molecular brightness, MB, are grouped into the size categories small (blue symbols) and large (green symbols).

#### Aggregate Category Characterization—Size, Concentration, and Backbone Conformation

The evolution of aggregate category sizes with time is monitored by determining the *R*_h_, as outlined in eq 1 (Experimental Section). Figure 6a illustrates the comparison of *R*_h_ values for the category of small and large aggregates over a seven-week period. Initially, the aggregates show an *R*_h_ of 62(26) nm derived from the mean and standard deviation of τ_d_ of all bursts belonging to the category small. The obtained value is in agreement with the previously determined FCS results of freshly prepared samples. During the course of one week, the *R*_h_ of the category of small aggregates decreased slightly to 55(22) nm, possibly indicating compaction. In contrast, the aggregates of the large category showed a slight increase of *R*_h_ of 283(183) and 315(216) nm after 1 and 7 weeks, respectively. The value of the larger *R*_h_ is consistent with the aggregate sizes observed by microscopy in Figure 3, taking into account the potential effects of deposition and the assumption of spherical particle shapes used in FCS modeling.

**Figure 6:**
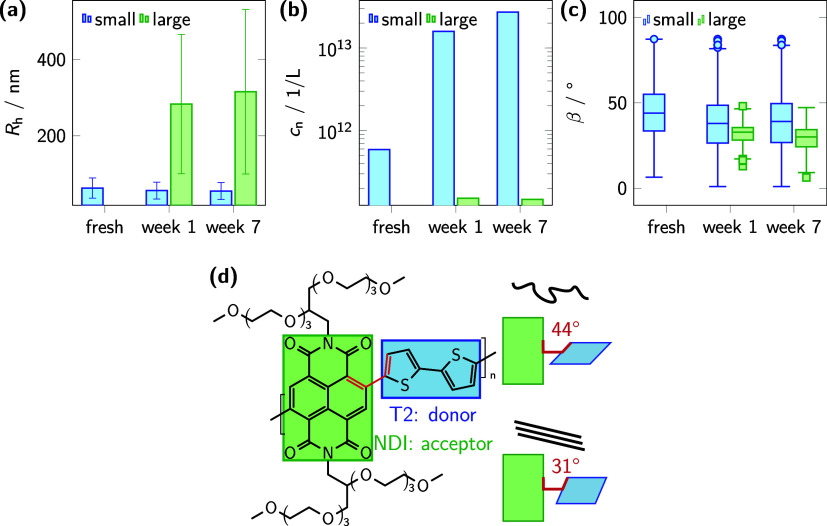
Time-dependent aggregate category characterizations in AS solutions by using smFS. (a) Time dependence of the mean *R*_h_ (bars) and standard deviation (error bars) of aggregates categorized as small (green bars) and large (blue bars). (b) Time dependence of the number concentration *c*_n_ of the aggregate categories small and large. (c) Box-car plots of the distributions of the angle β, for small and large categorized aggregates. (d) Structure of P­(EO-NDIT2)^28^ showing schematically the torsion angle (θ) between NDI and T2 units and the proposed reduction upon aggregation as provided by the DFT modeling of methylated trimers.^29^

In addition to size information, smFS allows us to monitor the number concentrations (*c*_n_) of each category based on eq 3. As depicted in Figure 6b, the concentration of small aggregates increased significantly during the first week as molecularly dissolved chains self-assemble into aggregates. Between one and seven weeks of aging, the concentration of small aggregates remained almost constant, indicating that the aggregation equilibrium was already reached within the first week, which is consistent with observations from UV/vis spectroscopy in Figure 1c. Initially, the category of large aggregates was not detected in the fresh sample, but these aggregates were formed within the first week. Following this initial formation period, their concentration remained constant, showing no further increase after seven weeks or even after sixty weeks. Given the solution’s mass concentration (*c*_m_) of 0.02 g L^–1^ and the polymer’s molar mass (*M*_n_), the expected *c*_n_ for individual chains is approximately 2.7 × 10^17^ chains/L. For the aggregate categories, the measured concentrations were approximately 1 × 10^13^ aggregates/L for the small and 1.5 × 10^11^ aggregates/L for the large aggregate category.

To investigate possible structural differences between the classified aggregates, we analyzed the emission fluorescence anisotropy (*r*) of each aggregate. *r* is capable of revealing the angle between the absorption and emission dipole moment (β), which has been previously used to study exciton migration within single conjugated polymers.^58^ Exciton migration leads to a decrease in both emission energy and *r*.^59^ The maximum value of *r* = 0.4 occurs when β = 0°, indicating that the emission and excitation dipoles are collinear. As the exciton migrates further from its creation site, the angle β will vary accordingly, with a maximum possible value of 90° (*r* = −0.2). While for open-coil polymer conformations, these variations will occur in a random manner with distributions centered around 0°, structural confinements may lead to average values distinct from 0° as shown in case of cocrystallization or π–π directed coassembly of conjugated donor–acceptor polymers.^60,61^ Hence, changes in β are capable of revealing changes in structural arrangements and local packing of polymer chains.^37,62^ The experimental results for the β values of fresh samples, both in AS and CB, showed an initial low anisotropy, with a mean value of β = 44°. With time, the mean β value decreased to 38° for the category of small aggregates, while the large aggregates appearing displayed an even lower, time-independent value of 31° (Figure 6c). Given the relatively low photon number per molecule, particularly for the category of small aggregates, the resulting widths of the distributions do not permit detailed interpretation. Interestingly, the mean values are comparable to torsion angles between NDI and T2 comonomers reported in DFT calculations (44°)^29^ and grazing angle infrared (IR) spectroscopy results on highly aggregated films of P­(OD-NDIT2) (38°).^63^

Initially, it is worth considering the possibility that aggregation-related electronic states of the same segment might already display different anisotropies. To investigate this, we compared our results to DFT calculations of the CT state of isolated chains and small aggregates.^29,48^ For isolated chains of P­(OD-NDIT2), the transition dipole moments of absorption and emission can be considered collinear.^48^ Even as CT excitation states of the chain are not confined to a single monomer and may differ between localized and delocalized excited states,^48^ the delocalization is constrained by structural relaxations, leading to a short finite segmental length of three to four repeat units.^64^ For dimers of P­(OD-NDIT2), time-dependent density functional theory (TDDFT) calculations revealed that the perpendicular component of the transition dipole moment is indeed related to the degree of exciton delocalization, the observed red-shift, as well as the alignment of the chains and structural segregation. However, the changes in polarization were found to be comparably small and could not describe the large β values observed in our work.^48^

Hence, we attribute the enhanced emission depolarization of the initial aggregates (β = 44°) to intersegmental energy transfer within the aggregate, by which an exciton moves to neighboring segments of a different crystallite with different orientations of transition dipole moments. With time, the mean β angle of the small aggregates decreased from β = 44° to 38°, suggesting a slow secondary process of restructuring. This process might be related to backbone planarization, a reduction of the torsion angle between NDI and T2 units, which has been predicted for P­(OD-NDIT2) dimers.^29,48^ Segmental structures, such as torsion angles and twist-angles, have been previously shown to play an important role in internal aggregate order.^37,65^ Interestingly, the larger aggregates, which only appear with time, display an even higher anisotropy (β = 31°), leading to the conclusion that these aggregates might form from smaller structural distinct aggregates with an enhanced (or even selective) tendency for further aggregation.

### Aggregates in CB Solutions

#### Burst Selection Criteria

In contrast to the aggregation in AS, which reaches equilibrium within a few days, aggregation in CB is a slow process that takes several months, as previously shown in Figure 1c. For comparison to our previous findings in AS, we examined samples in CB at various stages by using smFS.

#### Aggregate Category Characterization—Size, Concentration, and Backbone Conformation

Figure 7 presents a series of smFS data that allow us to follow the time dependence of aggregate formation in CB, as characterized by the molecular parameters τ_d_ and MB. In addition to the previous categories of small (blue symbols) and large (green symbols) aggregates, we introduced a new category of huge aggregates (red symbols), characterized by τ_d_ values that significantly exceed those observed in AS, as given in the Experimental Section. Figure 8 illustrates the time-dependent evolution of these three identified categories with respect to *R*_h_, *c*_n_, and β. The hydrodynamic radius in Figure 8a shows that within the first week, only the category of small aggregates are present, with an *R*_h_ of approximately 74(29) nm, which again displays the mean and the standard deviation of the category and is consistent with the sizes found by FCS of 65(27) nm. It is further comparable to initial aggregates of AS with 62(26) nm, as determined by single-molecule BAS. After seven weeks, aggregates of the category large with an *R*_h_ of 295(75) nm appeared, similar to those in AS with an *R*_h_ of 300(190) nm. Simultaneously and in contrast to AS, aggregates of the category huge emerged too, initially exhibiting an *R*_h_ of 1.3(0.5) μm. After 95 weeks, the majority (90%) of these aggregates displayed an *R*_h_ of 2.0(1.0) μm with a few even larger aggregates of an approximate mean *R*_h_ of 12 μm. The heterogeneity of aggregates within the categories, especially that of the huge category, becomes very apparent and is clearly visible in Figure 7.

**Figure 7:**
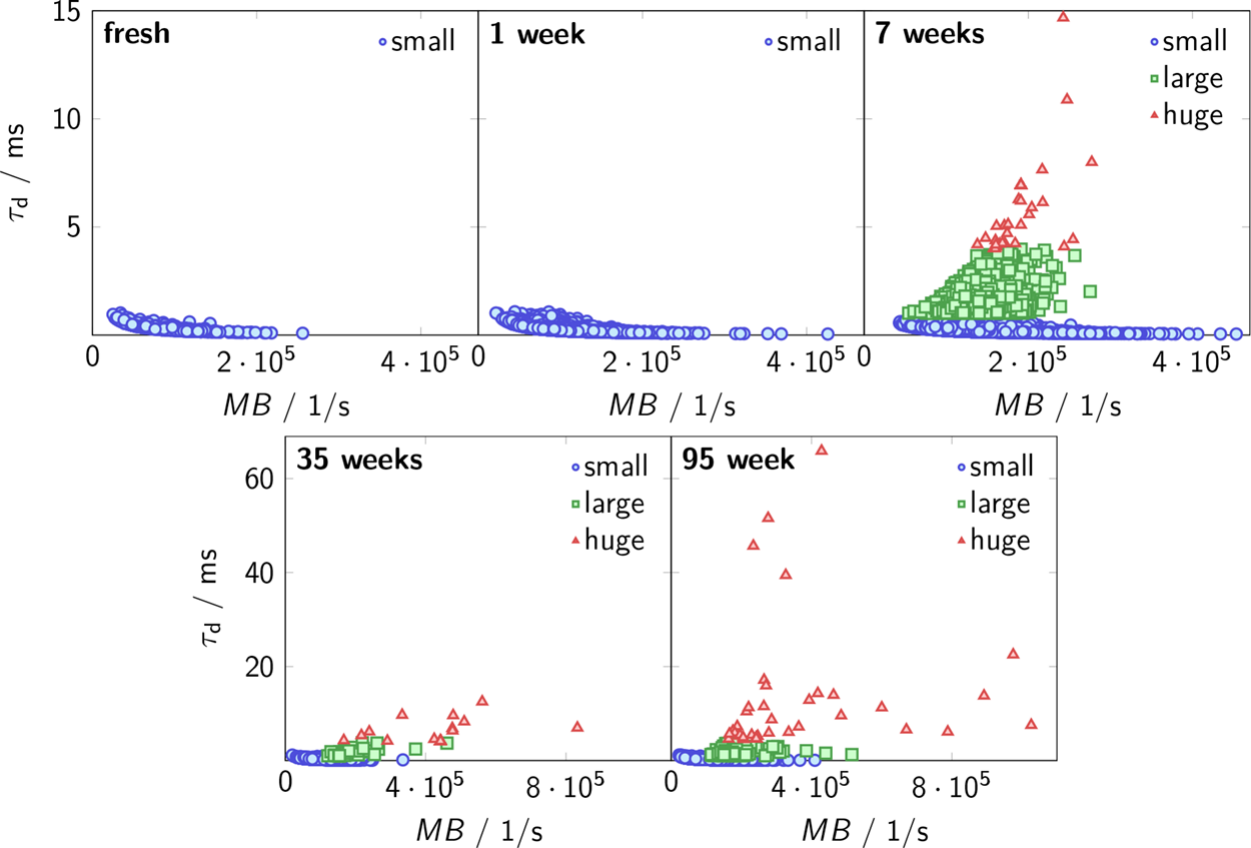
Time-dependent single-aggregate characteristics in CB solutions: results of the discriminant analysis of aggregates regarding diffusion time, τ_d_, and molecular brightness, MB, are grouped into the size categories small (blue symbols), large (green symbols), and huge (red symbols). Note the increased diffusion time scales (*y*-axis) for the lower graphs.

**Figure 8:**
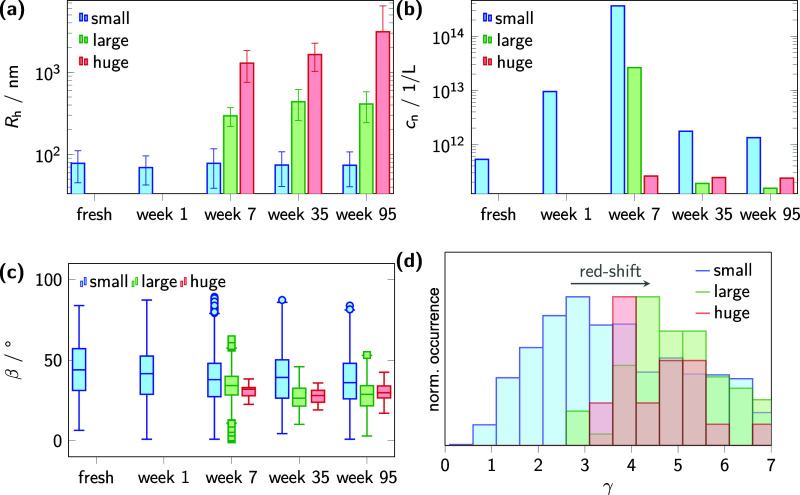
Time-dependent aggregate category characterizations in CB solutions using smFS. (a) Time dependence of the mean *R*_h_ (bars) and standard deviation (error bars) of aggregates categorized as small (green bars), large (blue bars), and huge (red bars). (b) Time dependence of the number concentration *c*_n_ of the aggregate fractions: small, large, and huge. (c) Box-car plots of the distributions of the angle β, for small, large, and huge categorized aggregates. (d) Histograms of the spectral shift analysis parameter γ for the differently categorized aggregates in an aged sample, see Figure 4.

Concerning concentrations displayed in Figure 8b, we found the *c*_n_ of the category of small aggregates to be initially 5 × 10^11^ L^–1^ in the fresh sample and to increase to a maximum value of 4 × 10^14^ L^–1^ after an aging time of seven weeks. After the course of 35 and 95 weeks, the *c*_n_ then decreases to 1 × 10^12^ L^–1^. The category of large aggregates, which first appeared at the seven-week measurement, have an initial *c*_n_ of 2 × 10^13^ L^–1^, but the concentration strongly declined to a final value of approximately 2.4 × 10^11^ L^–1^ by 35 weeks and remained constant thereafter. These final concentrations are 10-fold smaller than those for AS samples. Meanwhile, the category of huge aggregates, which are also first detected at seven weeks, equilibrate at the *c*_n_ of 1.7 × 10^11^ L^–1^, which is almost the concentration of the category of large aggregates for AS.

Regarding the fluorescence anisotropy, *r*, of the aggregates, the obtained mean values for β detected in CB are consistent with the findings in AS (see Figure 6c) and are shown in Figure 8c. Small aggregates exhibit a mean β of 44° in the fresh sample, which decreases to 38° from week seven onward. At seven weeks, when the categories of large and huge aggregates are first detected, the category of large aggregates shows a mean β of 34°, while aggregates of the category huge display a mean β of 32°. This angle decreases further to a range of 27–30° by 35 weeks, after which it remains constant, with no further change observed up to 95 weeks. Assuming a direct relationship between β and alignment of crystalline domains or the planarization of the monomeric subunits, these findings suggest structural differences between the aggregate categories regarding chain alignment and compaction, depending on their size.

#### Spectral Analysis of Aggregate Categories

By combining the smFS BAS with the previously described optical filtering (see Figure 4a), we are able to establish a more pronounced association between the red-shift and aggregate size.

As shown in Figure 8d, the distribution of the red-shift intensity ratio, γ, for the category of small aggregates peaks around 3.1, which is the mean γ of the background as discussed in Figure 4b. In contrast, the categories of large and huge aggregates are both significantly red-shifted with a most probable value of γ of 4.1 and 4.6, respectively.

The correlation between a reduction of the mean β-value and the distinct spectral red-shift hints toward local chain rearrangements within the aggregates which affect exciton dynamics between different CT-states leading effectively to a reduction of the 0–0 transitions in favor of 0–1 and 0–2 transitions.^37,65^ The rearrangements occur with time and can be associated with aggregates of sizes at and above 300 nm. Interestingly, no significant difference regarding the spectral shift is found between the categories large and huge. We can understand the optical features associated with red-shifted peaks in the absorption and fluorescence spectra, as previously assigned^29^ and illustrated in Figure 1, to indicate aggregates with higher internal order. This enhanced order is accompanied by a closer proximity of the chains due to π–π stacking, potentially increasing the compactness of the aggregates.

In summary, our single-molecule studies revealed solvent-dependent aggregation characteristics of P­(EO-NDIT2), including two classes of aggregates. In the nonaggregated state, the molecules exhibited a small size (*R*_h_ = 7 nm) and a low fluorescence emission per molecule (MB = 80 counts/s per molecule). With time, aggregates of different sizes formed: AS-induced aggregates were found in the size ranges of 55–65 nm and approximately 300 nm, while the slower aggregation process in CB added a third category of aggregates in the size range of up to 12 μm. Notably, the concentrations of the smaller aggregates consistently exceeded those of the larger aggregates. While the size of the aggregates within category huge continues to increase over an extended period of 95 weeks, the *c*_n_ of small and large aggregates decreases without a corresponding increase in *c*_n_ for the category of huge aggregates. This leads us to conclude that, in CB, smaller aggregates dissolve, favoring the growth of larger aggregates or possibly form supramolecular structures detectable as huge aggregates.

The increased MB of the aggregates ranging from 1000 to 100,000 counts/s per molecule allowed us to examine the spectral and anisotropic emission of the single aggregates. We found the *r* of the initial aggregates to be the lowest and comparable to AS measurements. The respective average β value was 44° and is again interpreted as the energy transfer between segments of differently oriented crystalline domains. In contrast, later appearing aggregates display an enhanced red-shift of the emission and an increased *r*, which was further linked to an increase in size. The increased red-shift may be linked to the growth of crystalline domains and enhanced intersegmental interactions that lead to a reduction of the 0–0 transitions in favor to 0–1 and 0–2 transitions. Likewise to the latter, the increase of *r* can be interpreted as a mutual alignment of the stacked domains. The increased order and local packing might be linked to preceding structural changes of the polymer’s torsion angle between NDI and T2 units (backbone planarization). In addition, progressive chain organization may also lead to a selective addition of chains or smaller aggregates, which results in size growth and significantly enhanced anisotropy and lower values for β of 34° and 32° for the large and huge aggregates, respectively.

## Conclusions

The combination of optical spectroscopy with FCS and smFS methods enables the detailed in situ characterization of P­(EO-NDIT2) aggregates. Time-dependent polymer chain self-assembly into aggregates at room temperature after thermal annealing of the polymer solutions in AS and CB was studied. Correlating the aggregates’ spectral properties with aggregate size, concentration, and fluorescence anisotropy, our results hint toward a size-dependent increase in alignment of crystalline domains accompanied by planarization of the backbone comonomer torsion angles. Our results are corroborated by complementary techniques, including optical microscopy, AFM, as well as DFT^29^ and grazing angle IR-spectroscopy.^63^

The findings presented herein contribute to a deeper understanding of aggregation processes auxiliary in ink preparation and storage in the field of printing organic electronic devices.

For P­(EO-NDIT2) in CB and AS solutions, the majority of aggregates, which were categorized as small, form immediately after thermal annealing and subsequent quenching to room temperature. They exhibit an average *R*_h_ of 60 nm and demonstrate a low MB. Time-dependent experiments reveal that, starting from molecularly dissolved polymer chains in solution, the concentration of the category of small aggregates reaches equilibrium within one week in AS solutions. Concurrently, the category of large aggregates emerges with an average *R*_h_ of 300 nm, a size consistent with atomic force microscopy (AFM) findings. In CB, the category of small aggregates with similar characteristics initially forms but partially dissolves again with time, favoring the growth of a third category of huge aggregates with a highly heterogeneous size distribution and an *R*_h_ up to 12 μm.

Our characterization of the aggregates included determining the angle between the absorption and emission dipole moment (β), which provides insights into the local chain arrangement. Our findings hint toward an increased order stemming from the alignment of crystalline domains accompanied by increased backbone planarity, which we suggest to result from denser chain packing and π–π-interactions, alongside with a pronounced spectral red-shift. This correlation connects the spectral features observed in absorbance and emission spectra to size and structural chain arrangements of aggregates.

The methodologies developed in this study can be extended to other fluorescence-active polymers, allowing the exploration of various nucleation and growth mechanisms, including temperature control, ultrasonic treatment, and current-induced nucleation. Such techniques could facilitate the production of solutions with precise aggregate size distributions and improved aggregate quality. This tunability has significant implications for optimizing ink shelf life, printability, and film microstructure, all of which directly impact the commercialization, performance, and stability of organic electronic devices.

Overall, this study provides valuable insights into the self-assembly of P­(EO-NDIT2) and sets the stage for future research aimed at controlling and optimizing polymer aggregation for advanced electronic applications.

## Experimental Section

### Synthesis and Characterization of P­(EO-NDIT2)

The P­(EO-NDIT2) sample used in this study was synthesized in-house following established procedures for side chain synthesis,^66^ monomer synthesis, and polymerization^28^ specified in Section S1.1 of the Supporting Information. Its *M*_n_ of 44 kg mol^–1^ was determined by end-group analysis^28^ in high-temperature nuclear magnetic resonance (NMR)-spectroscopy, as depicted in Figure S1 according to the end-group assignment by Shin et al.^28^

### Microscopy

The microscopy sample was prepared by drop-casting a sample solution with *c*_m_ = 0.08 mg mL^–1^. Aggregate AFM imaging was performed using a JPK Nanowizard II in tapping mode. The optical microscopy was conducted on a Leica device with 50× magnification, yielding a resolution of 9320 pixels/μm while applying ICR filters. For further details, see Section S1.2 of the Supporting Information.

### UV/Vis and Florescence Spectroscopy of Solutions

UV/vis and fluorescence spectra of the sample solution with *c*_m_ = 0.02 mg mL^–1^ were recorded in 10 mm cuvettes at room temperature, unless otherwise specified. The UV/vis measurements were conducted on a Cary 60 device by Agilent Technologies with optical glass cuvettes (Starna 21-SOG-10) in a spectral range from 900 to 300 nm with a 1 nm resolution. Absorption measurements of the polymer solutions were corrected by the background subtraction of the pure solvent. Fluorescence spectra were recorded on a Fluoromax 4 device by a HORIBA Jobin Yvon using suprasil quartz glass precision cuvettes (Hellma 101-QS), a xenon flash lamp, and a Czerny Turner monochromator. Light with a wavelength of the respective CT-absorption maximum of 600 and 740 nm was used for excitation. The sample spectra were recorded in a spectral range from 930 nm to 10 nm above the excitation wavelength with a 1 nm resolution. See Section S1.3 of the Supporting Information for further details.

### Fluorescence Correlation Spectroscopy (FCS) and Single-Molecule Fluorescence Spectroscopy (smFS)

#### Samples

The sample solutions with *c*_m_ = 0.02 mg mL^–1^ for FCS and smFS investigations were thermally annealed at 80 °C, followed by cooling to room temperature under ambient conditions. Data was collected by probing one sample in the solvents AS and CB at the fresh stage and after one and seven weeks. In CB, a second, comparable sample was probed at 35 and 95 weeks.

#### Setup

The measurements were conducted on a home-built confocal microscope setup. For polarization-sensitive experiments, the emitted photons were split by a polarizing beam splitter into a parallel (p-pol) and a perpendicular (s-pol) polarization component, as displayed in Figure 2a. Spectral shifts of the aggregate emissions were investigated using spectral filters, as Figure 4a shows. The technical description of the setup with its parts is specified in Section S1.4 of the Supporting Information.

The measurements were conducted at an excitation wavelength of 635 nm which lies within the CT band of the polymer. Using unbound Atto655 and Atto647N dye molecules (ATTO-TEC GmbH, Germany), the waist of the confocal volume, lateral waist of the confocal volume (ω_0_), was determined to be 424 and 434 nm for AS and CB solutions, respectively. The differences arise from different refractive indices.

#### Determination of *R*_h_, *c*_n_, and β

The *R*_h_ of polymers in the solution was obtained using the Stokes–Einstein relationship:

$$R_{h}=\frac{k_{B}T}{6\pi \eta D}$$ 1

with the Boltzmann constant (*k*_B_), the absolute temperature (*T*), the solvent viscosity (η), and diffusion coefficient (*D*). The latter is derived from the equation:^67^

$$D=\frac{\omega_{0}^{2}}{4\cdot \tau_{D}}$$ 2

with the lateral waist of the confocal volume (ω_0_) and the ensemble-averaged dwell time in the confocal volume, τ_D_.

The value of τ_D_ is either determined by FCS measurements using established standard curve fitting procedures as explained in the Supporting Information, Section S1.5, or derived from the average of the apparent dwell times, ⟨τ_d_⟩, of all fluorescence bursts assigned to one category. This discriminant analysis of aggregates into categories was performed by comparing the 2D-plot of τ_d_ vs MB of the fresh samples with the consecutive plots of the aged samples. In the case of AS, the boundary between early and late aggregates, categorized as small and large, was found to follow the relation τ_d_ = 100 cts/MB. While in the case of CB, the boundaries were τ_d_ = 1 ms between the categories called small and large and τ_d_ = 4 ms between the categories called large and huge. For ⟨τ_d_⟩, a correction factor (*f*) was attributed with τ_D_ = *f*⟨τ_d_⟩, which was experimentally determined by comparing the FCS result with ⟨τ_d_⟩ of freshly prepared samples. Details about the burst selection can be found in the Supporting Information, Section S1.6.

The aggregate concentration of each category, *c*_n_, was determined by the equation^42^

$$c_{n}=\frac{\epsilon \langle \tau_{d}\rangle }{\pi \omega_{0}^{3}}$$ 3

where the burst encounter rate (ϵ) describes the number of bursts of each category detected per time unit.

The distributions of the angle between the absorption and emission dipole moment (β) of selected categories were retrieved from the individual β-values of the selected aggregates obtained by the equation^67^

$$\beta =\arccos\left(\sqrt{\frac{1+5r}{3}}\right)\frac{180}{\pi }$$ 4

where the molecular fluorescence anisotropy (*r*) is defined in Section S1.6 of the Supporting Information.

## Supplementary Material



## Notes

Synthesis and characterization of P­(EO-NDIT2); AFM and light microscopy; UV/vis and fluorescence spectroscopy; fluorescence microscope; and data analysis of FCS and smFS experiments (PDF)
