# Supplementary material for: Time-Dependent Structure Assessment of Conjugated Polymer Aggregates in Solution by Single-Molecule Fluorescence Spectroscopy
Source: Macromolecules. 2026 Jan 21;59(3):1740–51. doi: 10.1021/acs.macromol.5c02031 (PMC12895533; doi:10.1021/acs.macromol.5c02031)
Supplement: Supplementary file 1 [file ma5c02031_si_001.pdf]

Supporting Information: Time-dependent structure assessment of conjugated polymer aggregates in solution by single-molecule fluorescence spectroscopy

Esther Schäfer,[†] Michael Sommer,[*,†] and Maria Ott[*,‡]

†*Institut für Chemie, Technische Universität Chemnitz, Professur Polymerchemie, Straße der Nationen 62, 09111 Chemnitz, Germany*

‡*Institute of Biochemistry and Biotechnology, Martin Luther University Halle-Wittenberg, Protein Biochemistry, Kurt-Mothes-Str. 3, 06120 Halle (Saale), Germany*

E-mail: michael.sommer@chemie.tu-chemnitz.de; maria.ott@bct.uni-halle.de

Contents

S1 Methods **3**

S1.1 Synthesis and characterization of P(EO-NDIT2) 3

S1.2 AFM and Light Microscopy 4

S1.3 UV/vis and fluorescence spectroscopy 5

S1.4 Fluorescence Microscope 5

S1.5 Data analysis of FCS experiments 6

S1.6 Data analysis of smFS experiments 7

S2 Figures **9**

S3 References **11**

S1 Methods

S1.1 Synthesis and characterization of P(EO-NDIT2)

The poly{[*N,N′*-2,3-bis(methoxy(triethylenoxide))propane-naphthalene-1,4,5,8-bis(dicarboximide)-2,6-diyl]-*alt*-5,5′-(2,2′-bithiophene)} (P(EO-NDIT2)) sample used for investigations in this work was synthesized following procedures for side chain synthesis,[1] monomer synthesis, and polymerization[2] as documented elsewhere. End group analysis[2] of the high temperature nuclear magnetic resonsce (NMR)-spectrum in Figure S1 at 120 °C conducted in an 250 mHz Bruker NMR in $C_2D_2Cl_4$ allows for determination of degree of polymerization (DP_n) by

$$DP_n = \frac{I_{\text{backbone H}}}{\frac{1}{2}I_{\text{engroup H}}} \tag{1}$$

with the intensity of the respective signals.

For the calculation of DP_n and molar mass (M_n), we use the two backbone H of naphthalene-1,4,5,8-bis(dicarboximide) (NDI). As the observed endgroups are methylated NDI, methylated 2,2′-bithiophene (T2) and just T2, the intensity of one H of each is taken into account. The signal at 7.03 ppm is a combination of two H from different T2 endgroups and the backbone signals satellite.

$$DP_n = \frac{I_{\text{NDI 2H,8.83 ppm}}}{I_{\text{NDI H,8.64 ppm}} + I_{\text{T2 H}_{\text{f,H}}\text{,7.03 ppm}} + I_{\text{T2 H}_{\text{e,Me}}\text{,7.03 ppm}}} \tag{2}$$

$$= \frac{I_{\text{NDI 2H,8.83 ppm}}}{I_{\text{NDI H,8.64 ppm}} + I_{\text{7.03 ppm}} - I_{\text{satellite,7.71 ppm}}} \tag{3}$$

With the resulting DP_n of 38 the molar mass of 1161.34 g mol^{-1} per repeat unit, the M_n of 44 kg mol^{-1} was determined. Choosing different signals for the calculation results in slightly varying values.

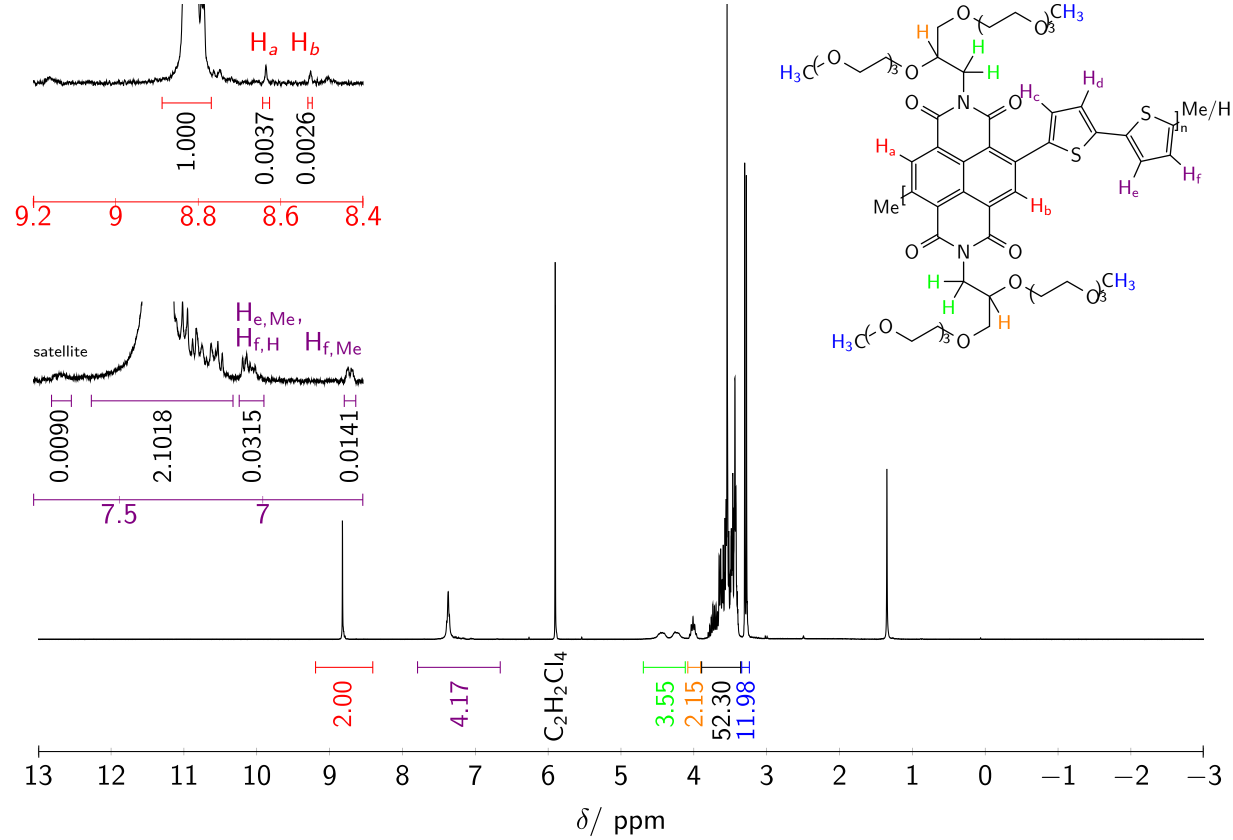

Figure S1: ^{1}H-NMR of P(EO-NDIT2) with methyl-endgroups at 120 °C in $C_2D_2Cl_4$ with assignment of signals by color-coding and magnifications showing the endgroup-proton signals and their respective intensities in comparison to the predominent in-chain proton signals. The endgroup evaluation brings forth a M_n of approximately 44 kg mol^{-1}.

S1.2 AFM and Light Microscopy

Imaging of the aggregates was conducted by atomic force microscopy (AFM) on a JPK Nanowizard II and Light Microscopy utilizing interference contrast (ICR) filters on a Leica device with 50x magnification yielding a resolution of 9320 pixel/µm. The images were evaluated using the software Gwyddion.

For sample preparations the approx. 1x1cm^2 Si-wafers were ultrasonicated in acetone and isopropanol for 10 min each, heated to 150 °C on a metal plate and cleaned by a CO_2/ice crystal gun in short proximity doing a zig zag motion, pointing the gun on the substrate at an angle smaller 90°.

10 µL of the sample solution of the mass concentration (c_m)=0.08 mg mL^{-1} were drop cast onto the cleaned substrates. In a vacuum of 70 mbar the solvent was evaporated.

S1.3 UV/vis and fluorescence spectroscopy

ultraviolet-visible (UV/vis) spectra were recorded in transmission on a Cary 60 device by Agilent Technologies in 10 mm cuvettes made of optical glass (Starna 21-SOG-10). Fluorescence spectra were recorded on a Fluoromax 4 device by HORIBA Jobin Yvon in 10 mm suprasil using precision cuvettes (101-QS) made of quartz glass, a xenon flash lamp, and a Czerny Turner monochromator. Light with a wavelength of the respective charge transfer (CT)-absorption maximum was used for excitation. The measurements were conducted at room temperature if not specified differently.

S1.4 Fluorescence Microscope

The fluorescence correlation spectroscopy (FCS) and single-molecule fluorescence spectroscopy (smFS) experiments were conducted with a home-built confocal microscope equipped with a 60X water immersion objective (UPLSAPO60XW/1.20, Olympus K.K., Japan) and a configuration described elsewhere.[3] Measurements were performed at an excitation wavelength of 635 nm, which is well within the CT band of the polymer. A pulsed laser was used, operating at 80 MHz, with a pulse width of 1 ps and an average laser power of 2.5 µW. A beamsplitter cube (Melles Griot) combined with various sets of long and short pass filters (LP640, SP775, LP775, SP842 and LP834, all from Semrock Inc.) were used to achieve polarization and spectral-sensitive measurements. The single-photon detection was realized by two single-photon avalanche diodes (SPCM-AQRH-14-TR, Excelitas Technologies Corp.) in combination with a single-photon counting board (TimeHarp260, TTTR mode, Picoquant GmbH, Berlin). All measurements were conducted in 10 µm depth and corrected for apparent solution-dependent effects on the confocal volume using fluorescent dyes as Atto 655 and Atto 647N (Atto-Tec GmbH) with known diffusion coefficients for reference mea-

surements. Data processing and analysis were performed using IgorPro 8 (Wavemetrics), a comprehensive software package for data analysis and visualization.

S1.5 Data analysis of FCS experiments

The cross-correlation functions, $G(\tau)$, were calculated from the photon arrival times of two detection channels using well-established routines.[4] τ is the lag time of the correlation procedure. In case of pure diffusion, the correlation function of a heterogeneous sample can be described as a weighted sum of its components:[5]

$$G(\tau) = 1 + \frac{\sum_i N_i B_i^2 g_i(\tau)}{(\sum_i N_i B_i)^2} \tag{4}$$

where N_i is the average number of molecules of species i in the confocal volume and B_i is its molecular brightness. B_i is directly related to the the cross-sectional area of absorption and the quantum efficiency of the molecule. $g_i(\tau)$ describes the time dependence of the correlation decay of a single species and can be written as[5]

$$g_i(\tau) = \left(1 - \left(\frac{\tau}{\tau_{\mathrm{D},i}}\right)\right)^{-1} \left(1 - \frac{1}{S^2}\left(\frac{\tau}{\tau_{\mathrm{D},i}}\right)\right)^{-1/2} \tag{5}$$

were S is a fixed parameters describing the 3D-shape of the elliptical focus volume, and $\tau_{\mathrm{D},i}$ refers to the average molecular dwell time within the focus volume and is directly related to the translational diffusion coefficient, D_i:[5]

$$D_i = \frac{{\omega_0}^2}{4\tau_{\mathrm{D},i}} \tag{6}$$

The width of the confocal volume, ω_0, was determined for each solution by reference measurements of the red-emitting fluorophore Atto 655 with known diffusion coefficient.[6] Within the validity of the Stokes-Einstein relation for sphere diffusion, D_i can be related to an apparent

hydrodynamic radius, R_h:[7]

$$R_{\mathrm{h},i} = \frac{k_\mathrm{B}T}{6\pi\eta D_i} \tag{7}$$

where k_B is the Boltzmann constant, T the temperature and η the solvent's viscosity retrived from standard tables with 1.009 mPa s and 0.744 mPa s for anisole (AS) and chlorobenzene (CB), respectively. For a comprehensive comparison of the characteristic decay profiles of different samples, $G(\tau)$ of eq. 4 can be normalized with respect to the total number of molecules in the confocal volume by the equation

$$G_\mathrm{n}(\tau) = 1 + G(\tau)\left(\sum_i N_i B_i\right)^2 \tag{8}$$

For the characterization of fresh samples, a two-component fit was conducted, assigning the fast decaying component to non-aggregated polymer molecules and the slow decaying component to aggregates. Aged samples were too heterogeneous in size to be characterized by FCS methods.

S1.6 Data analysis of smFS experiments

In order to analyze the photon bursts of single aggregates, the photons of each each aggregate needed to be identified and isolated from the entire set of photons. Therefore, the first step was to eliminate the contributions of non-aggregated chains, which appeared as a constant background due to their comparably low molecular brightness and high number concentrations (an average of approx. 30 molecules within the confocal volume). In contrast, the low concentrations of the aggregates (less than 1 molecule within the confocal volume) combined with a much higher molecular brightness of the aggregated molecules lead to large and well-separated intensity fluctuations (see Figure 2, main text). The photon time traces were then analyzed by selecting photons of either detection channel using a threshold criterium of 50 000 photons/s and combining them into single bursts using routines established by Wägele *et al.*[8] The burst photons of aggregates with a minimum number of 20 photons,

were then characterized by their photon number n, passage time τ_d (which was quantified by the standard deviation of the burst photon arrival times), their molecular brightness $MB = n/\tau_\mathrm{d}$, and their apparent anisotropy $r = (n_{||} - Gn_\perp)/(n_{||} + 2Gn_\perp)$, with $n_{||}$ and $n_\perp$ being the number of photons detected in the parallel and perpendicular detection channel, respectively. The factor G was used to correct for channel related differences of the detection efficiencies.[5] To exclude possible fluence-dependent effects on fluorescence anisotropy (r), we performed a control experiment on the 35 week sample in CB at excitation powers of 2.5 µW and 25 µW. No significant difference in the mean values of the angle between the absorption and emission dipole moment (β) was observed, confirming that the reported values are not influenced by the excitation power within this range. Grouping of aggregates into categories allowed to calculate a mean passage time, τ_d, which was used to determine the concentration and the average size of the grouped molecules. The size was determined by the use of equation 7 and relating τ_D to τ_d via a factor f, which was derived by comparison of the single-molecule burst data to the correlation function of a freshly heated sample (τ_d=$f\tau_\mathrm{D}$).

S2 Figures

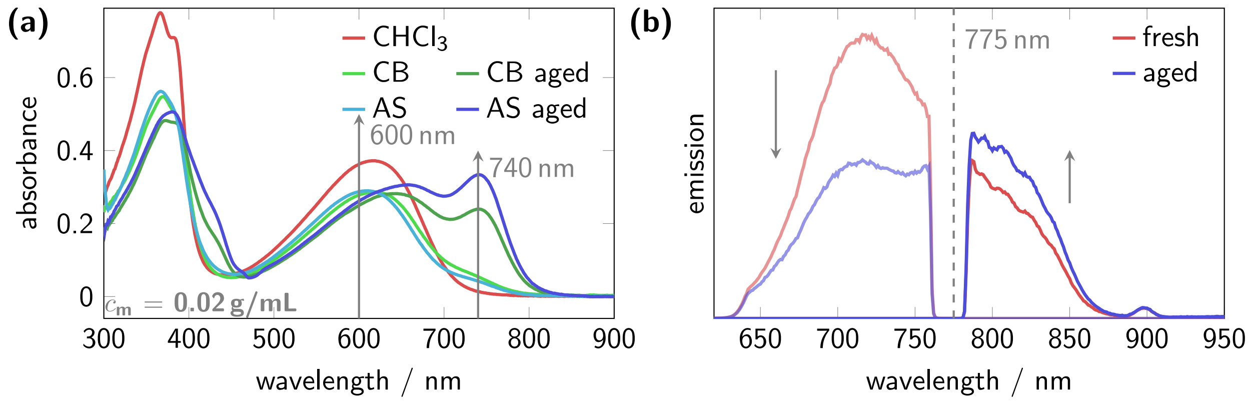

Figure S2: Optical Spectroscopy on P(EO-NDIT2) solutions in different solvents. (a) UV/vis spectra showing low aggregation after sample preparation at 80 °C and high aggregation in aged solutions, indicated by the CT band centered at 740 nm. Solutions in chloroform ($CHCl_3$) neither show aggregation past cooling to −18 °C nor aging at ambient condition for two month. $CHCl_3$ is thus considered to serve well for the preparation of mother solutions. (b) Fluorescence spectra for freshly heated and aged solutions in CB with the application of optical filters at 775 nm for photon discrimination between non-aggregated chains and aggregates.

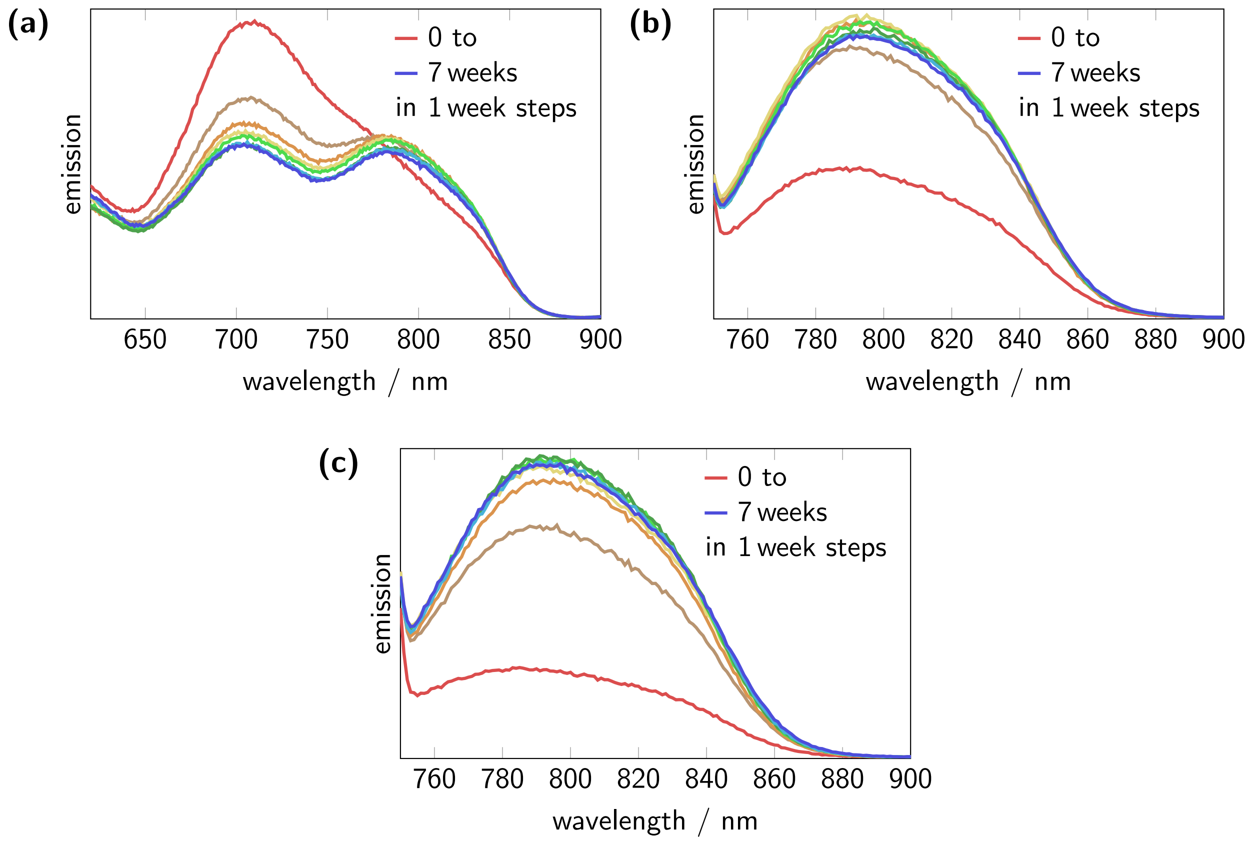

Figure S3: Fluorescence spectroscopy on P(EO-NDIT2) for solution aging at different excitation wavelengths. (a) Fluorescence spectra of aging process in AS. (λ_{exc}=600 nm) (b) Fluorescence spectra of aging process in AS. (λ_{exc}=740 nm) (c) Fluorescence spectra of aging process in CB. (λ_{exc}=740 nm)

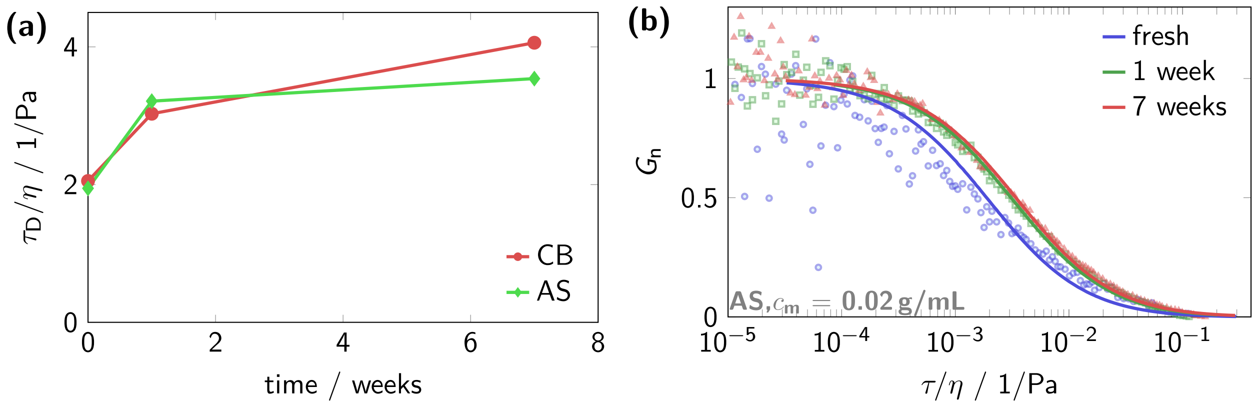

Figure S4: FCS results with (a) showing the time-dependent increase and equilibration of the aggregate's average passage times in AS and CB normalized over the solvent viscosity (η) for comparison across solvents. Figure 2c (main text) shows the correlation functions (empty symbols) in CB, (b) in AS from which the dwell time (τ_D) were determined by fitting (lines).

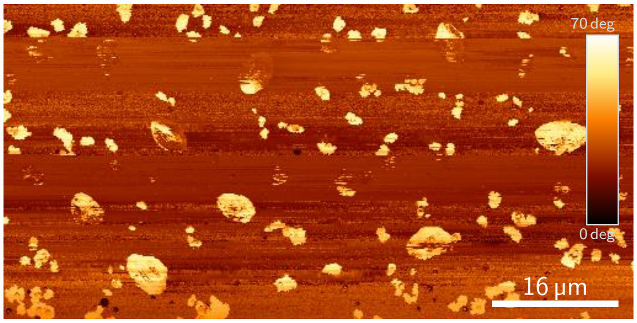

Figure S5: AFM phase image corresponding to Figure 3 (main text) displaying the phase difference between wafer and small aggregates, while their observation in the height image is not possible.

S3 References

References

(1) O'Driscoll, L. J.; Welsh, D. J.; Bailey, S. W. D.; Visontai, D.; Frampton, H.; Bryce, M. R.; Lambert, C. J. Reversible Thermal Switching of Aqueous Dispersibility of Multiwalled

Carbon Nanotubes. *Chemistry – A European Journal* **2015**, *21*, 3891–3894, DOI: 10.1002/chem.201405804.

(2) Shin, Y.-h. *et al.* Synthesis and Aggregation Behavior of a Glycolated Naphthalene Diimide Bithiophene Copolymer for Application in Low-Level n-Doped Organic Thermoelectrics. *Macromolecules* **2020**, *53*, 5158–5168, DOI: 10.1021/acs.macromol.0c00657.

(3) Roos, M.; Ott, M.; Hofmann, M.; Link, S.; Rössler, E.; Balbach, J.; Krushelnitsky, A.; Saalwächter, K. Coupling and Decoupling of Rotational and Translational Diffusion of Proteins under Crowding Conditions. *Journal of the American Chemical Society* **2016**, *138*, 10365–10372, DOI: 10.1021/jacs.6b06615.

(4) Wahl, M.; Gregor, I.; Patting, M.; Enderlein, J. Fast calculation of fluorescence correlation data with asynchronous time-correlated single-photon counting. *Optics Express* **2003**, *11*, 3583, DOI: 10.1364/oe.11.003583.

(5) Lakowicz, J. R. *Principles of Fluorescence Spectroscopy*, 3rd ed.; Springer, Boston, MA, 2006; DOI: 10.1007/978-0-387-46312-4.

(6) Müller, C. B.; Loman, A.; Pacheco, V.; Koberling, F.; Willbold, D.; Richtering, W.; Enderlein, J. Precise measurement of diffusion by multi-color dual-focus fluorescence correlation spectroscopy. *EPL (Europhysics Letters)* **2008**, *83*, 46001, DOI: 10.1209/0295-5075/83/46001.

(7) Einstein, A. Über die von der molekularkinetischen Theorie der Wärme geforderte Bewegung von in ruhenden Flüssigkeiten suspendierten Teilchen. *Annalen der Physik* **1905**, *322*, 549–560, DOI: 10.1002/andp.19053220806.

(8) Wägele, J.; De Sio, S.; Voigt, B.; Balbach, J.; Ott, M. How Fluorescent Tags Modify Oligomer Size Distributions of the Alzheimer Peptide. *Biophysical Journal* **2019**, *116*, 227–238, DOI: 10.1016/j.bpj.2018.12.010.
